# Effects of gp120 Inner Domain (ID2) Immunogen Doses on Elicitation of Anti-HIV-1 Functional Fc-Effector Response to C1/C2 (Cluster A) Epitopes in Mice

**DOI:** 10.3390/microorganisms8101490

**Published:** 2020-09-28

**Authors:** Rebekah Sherburn, William D. Tolbert, Suneetha Gottumukkala, Guillaume Beaudoin-Bussières, Andrés Finzi, Marzena Pazgier

**Affiliations:** 1 Infectious Disease Division, Department of Medicine of Uniformed Services University of the Health Sciences, Bethesda, MD 20814-4712, USA; rebekah.sherburn.ctr@usuhs.edu (R.S.); William.tolbert.ctr@usuhs.edu (W.D.T.); Suneetha.Gottumukkala.ctr@usuhs.edu (S.G.); 2 Centre de Recherche du CHUM, Montreal, QC H2X 0A9, Canada; guillaume.beaudoin-bussieres@umontreal.ca (G.B.-B.); andres.finzi@umontreal.ca (A.F.); 3 Département de Microbiologie, Infectiologie et Immunologie, Université de Montréal, Montreal, QC H3C 3J7, Canada; 4 Department of Microbiology and Immunology, McGill University, Montreal, QC H3A 2B4, Canada

**Keywords:** HIV-1, ADCC, inner domain (ID2) immunogen, non-neutralizing antibody response, dosing, fc-mediated effector functions, isotype

## Abstract

Fc-mediated effector functions of antibodies, including antibody-dependent cytotoxicity (ADCC), have been shown to contribute to vaccine-induced protection from HIV-1 infection, especially those directed against non-neutralizing, CD4 inducible (CD4i) epitopes within the gp120 constant 1 and 2 regions (C1/C2 or Cluster A epitopes). However, recent passive immunization studies have not been able to definitively confirm roles for these antibodies in HIV-1 prevention mostly due to the complications of cross-species Fc–FcR interactions and suboptimal dosing strategies. Here, we use our stabilized gp120 Inner domain (ID2) immunogen that displays the Cluster A epitopes within a minimal structural unit of HIV-1 Env to investigate an immunization protocol that induces a fine-tuned antibody repertoire capable of an effective Fc-effector response. This includes the generation of isotypes and the enhanced antibody specificity known to be vital for maximal Fc-effector activities, while minimizing the induction of isotypes know to be detrimental for these functions. Although our studies were done in in BALB/c mice we conclude that when optimally titrated for the species of interest, ID2 with GLA-SE adjuvant will elicit high titers of antibodies targeting the Cluster A region with potent Fc-mediated effector functions, making it a valuable immunogen candidate for testing an exclusive role of non-neutralizing antibody response in HIV-1 protection in vaccine settings.

## 1. Introduction

Despite decades of research, no licensed preventative or therapeutic HIV-1 vaccine is available. Antiretroviral therapy (ART) offers promising control of disease progression and limits transmission from HIV^+^ individuals; however, it comes with both a heavy financial burden and risk of severe side effects [1,2,3,4]. A preventative vaccine is desperately required but efforts to date have had limited success due to a number of viral and immune system obstacles. HIV has evolved extremely successful immune evasion mechanisms that greatly diminish the effectiveness of humoral responses induced with currently available HIV vaccine candidates [5,6]. There are two main mechanisms by which vaccine-induced antibodies are able to impact HIV infection: direct neutralization and through Fc-mediated effector functions. In recent years a number of monoclonal antibodies (mAbs) capable of broad virus neutralization (broadly neutralizing antibodies (bnAbs)) have been isolated, mostly from natural infection with the capability of neutralization of viruses across multiple clades to confer protection from infection when passively administered in effective doses [7,8,9]. Unfortunately, there is no vaccination strategy capable of inducing bnAbs at protective levels in any animal model or human trial [10]. Therefore, the focus has shifted to inducing antibodies capable of protective Fc-effector function against free virus or HIV-infected cells through mechanisms including antibody dependent cellular phagocytosis (ADCP) and antibody-dependent cellular cytotoxicity (ADCC) even though they display weak or no direct neutralization activity [11]. This group of antibodies lacking direct neutralization activity are referred to as non-neutralizing antibodies (nnAbs). There is strong evidence that conformational CD4 inducible (CD4i) epitopes within the Cluster A region are targets for Abs capable of potent ADCC without direct neutralization activity [12,13,14,15,16]. We characterized the binding sites of antibodies specific for the Cluster A region and mapped their epitope to a highly conserved region of CD4-triggered gp120 within constant region 1 and 2 (C1/C2) of the gp120 inner domain [17,18]. Our structural analyses revealed that the Cluster A region maps to the gp120 face directly involved in the contact with gp41 within the untriggered HIV-1 trimer and therefore its accessibility for antibody recognition is strictly dependent on the conformational state of the HIV-1 trimer post CD4-binding. Our and other data indicate that these strictly CD4-dependent targets are only exposed during certain windows in the HIV-1 replication cycle, primarily during the HIV entry process, when the Env trimer attaches to target cell CD4 and the conformational rearrangements required for fusion occur [19,20,21,22,23,24]. Exposure of Cluster A epitopes on HIV-infected cells is limited due to the low abundance of CD4 that is required to trigger Env trimers emerging at the infected cell surface [25]. However, these targets are detected in infected cell populations that retain some levels of CD4, [14,26,27,28], and are present during the process of cell-to-cell spread [29,30].

Non-neutralizing Ab (nnAb) responses to C1/C2 have been evaluated primarily in the context of natural infection as Cluster A Env sites are highly immunogenic and the Ab response to this region is detected in most infected individuals [13,14,31,32,33]. Interestingly, ADCC against this region has also been shown to be the major ADCC response in chronically infected individuals [13,26,31,34,35] and CD4i Abs with ADCC activity are known to be passively transferred in utero via placental transfer to babies born to HIV-infected mothers [36]; however, it is still unclear whether these responses are protective and/or beneficial [13,14,34,35,36,37,38]. One recent study has quantified the relative contribution of Fc-mediated effector functions against HIV-1 and showed that 25–45% of the humoral response to HIV-1 in humanized mice and to SHIV in infected macaques was Fc-mediated, revealing this process is likely vital for control of HIV-1 [39].

The recent ALVAC/AIDSVAX B/E RV144 vaccine trial in which modest protection from HIV-1 infection (31.2%) was observed, renewed interest in non-neutralizing epitope targets and stimulated research to understand the molecular basis for potent Env ADCC epitopes, including those to the C1/C2 region. The RV144 immunization regimen selectively induced a non- or weakly neutralizing response with highly coordinated FcR- functions [40,41], correlating with a decreased risk of HIV-1 infection in a blinded follow-up case-control study [40]. The array of mAbs elicited in the trial was narrow, with specificities elicited for V2 loop [42] and C1/C2 region epitopes [43,44,45]. While only ADCC mediated by V2-specific Abs was directly linked to protection, A32-like and V2-specific Abs synergized for effective ADCC [46]. ADCC mediated by anti-cluster A Abs in the RV144 trial was linked to the presence of a gp120 Phe43 filling residue, H375, in the main circulating CRF01_AE strain in Thailand. This residue predisposes Env to sample more “open” conformations, thereby sensitizing infected cells to ADCC [47]. Interestingly, the results from the recent RV144 follow up study, RV305, indicated that A32-like ADCC responses can be effectively boosted. Sera from RV305 subjects were capable of effective ADCC with increased potency and breadth compared to C1/C2 mAbs isolated from RV144 subjects [48,49]. Our structural analyses RV144 and RV305 mAbs confirmed their Cluster A specificity [50].

We previously developed an optimally designed minimal structural unit to display Cluster A epitopes in the absence of any other known neutralizing epitopes, an inner domain construct stabilized in the CD4-bound conformation known as ID2 [51,52]. ID2 has the potential to be an optimal immunogen/vaccine candidate for the selective induction of an ADCC antibody response to the Cluster A region without ‘complications’ of responses to other neutralizing and non-neutralizing epitope specificities. Our previous structural analysis revealed that ID2 optimally displayed Cluster A epitopes in the CD4-triggered conformation, and initial immunization studies in a BALB/c mouse model of ID2 with the adjuvant GLA-SE revealed a strong induction of antibodies capable of competing with prototype HIV-1 nnAbs A32 and N5-i5 [52]. Interestingly, this study also demonstrated that there was a significant difference in the elicitation of antibodies capable of mediating ADCC that was dependent on the adjuvant used when the route of administration and amount of protein was identical, indicating that the isotype repertoire elicited in response to immunization is likely an important determinant of Fc-mediated effector potential. Here, we describe how the ID2 immunogen dose affects the quantity and quality of the induced Cluster-A-specific immune response in immunized mice and show how this correlates with the effectiveness of sera antibodies in mediating Fc-effector functions against variable targets.

## 2. Materials and Methods

### 2.1. Immunogen Expression, Isolation and Preparation

ID2 was expressed and isolated as previously described [52]. Briefly, stable HEK293 cell lines containing the ID2 expression plasmid were cultured for 6–7 days before the collection of the supernatant and passage through a 0.22 µm filter. Media were then run over an N5-i5 affinity column, washed thoroughly with PBS and protein eluted with 0.1 M glycine at pH3. ID2 was analyzed via SDS-PAGE, dialyzed to PBS and sterile filtered before diluting to the appropriate concentration for a final injection amount of 0.25, 0.74, 2.22, 6.67, 20 or 60 µg. Immunizations were made up in 80 µl PBS with 20 µl GLA-SE adjuvant (stable oil-in water emulsion containing TLR-4 agonist developed by Infectious Disease Research Institute, Catalog # IDRI-GLA-SE) per dose added within an hour of injection.

### 2.2. Mice and Immunization Protocol

Female and male BALB/c mice were purchased from the Jackson Laboratory and cared for in accordance with the Association for the Assessment and Accreditation of Laboratory Animal Care International (AAALAC) standard. All procedures were approved by the University of Maryland IACUC committee. Mice aged 6–8 weeks were immunized with varying amounts of ID2 via the intraperitoneal route, as described in [52] at week 0, 2, 4 and 8. Blood was collected 2 weeks following each immunization and sera processed and stored at −20 °C until required.

### 2.3. Immunogen Specific ELISA

For the detection of ID2-specific antibodies in sera, an ELISA platform was utilized. 96-well Nunc Maxisorp plates (Millipore Sigma, Burlington, MA, USA) were coated with 50 ng per well ID2 in Tris-buffered saline (TBS) overnight at 4 °C. Plates were washed with 3× TBS + 0.05% Tween-20 before being blocked at room temperature for 2 h with TBS + 5% non-fat milk powder and 0.1% Tergitol. Sera from individual mice were diluted with blocking buffer in a 10-fold dilution starting at 1:100 and added to blocked plates before incubating overnight at 4 °C. Following 3 washes, the plates were probed with an alkaline phosphatase secondary antibody (Southern Biotech, Birmingham, AL, USA) against total IgG, IgG1, IgG2a, IgG2b, IgG2c, IgG3, IgA and IgM at 1:1000 dilution in blocking buffer for 1 h at 37 °C. Any unbound secondary antibody was then washed from the plate and the assay was developed using a Blue Phos Microwell Phosphatase Substrate System and stopped following 15 min incubation at room temperature using APstop Solution (Both Seracare Life Sciences, Milford, MA, USA). The plates were then read at 620 nm and the optical density recorded. All sera samples were measured for each individual mouse in triplicate. GraphPad Prism (Version 7.05, San Diego, CA, USA) was used to display the mean and SEM for all groups and used to calculate the Area under the Curve (AUC).

### 2.4. RFADCC and ADCC

Relative fluorescence antibody dependent cellular cytotoxicity assays were carried out as previously described, with minor adjustments [50,52,53]. Briefly, EGFP-CEM-NKR-CCR5SNAP cells were coated with one of three antigen targets: gp120_93TH057_ core, full-length gp120_93TH057_ or gp120_BaL_, all at 50 µg/mL. Cells were then incubated with pooled or individual sera (starting at 1:50 dilution), positive control A32 (starting at 10 µg/mL) or naïve pre-bleed sera over a 1:4 dilution range. Antibody-bound cells were then cultured with human PBMCs (Stemcell Technologies, Vancouver, BC, Canada) for 3 h at 37 °C before washing and fixing in 1% paraformaldehyde prior to analysis by flow cytometry using a BD LSR II special order instrument (BD, Franklin Lakes, NJ, USA). Data were analyzed using FlowJo (Version 10, Tree Star, Ashland, OR, USA)) and plotted using Graphpad Prism by normalizing data to the A32 positive control and naïve sera negative control to determine the percent lysis.

ADCC against ADA-Env-based NL4.3 GFP viruses containing intact (WT) or defective *nef* and *vpu* genes (N-U-) was carried out as previously described [54,55]. For the evaluation of antibody-dependent cellular cytotoxicity (ADCC), infected primary CD4+ T cells were stained with viability (AquaVivid; Thermo Fisher Scientific, Waltham, MA, USA) and cellular (cell proliferation dye eFluor670; Thermo Fisher Scientific, Waltham, MA, USA) markers and used as target cells. Overnight-rested autologous PBMCs were stained with another cellular marker (cell proliferation dye eFluor450; Thermo Fisher Scientific, Waltham, MA, USA). The primary CD4+ T cells and PBMCs were then incubated 20 min before being washed twice in complete RPMI (Thermo Fisher Scientific, Waltham, MA, USA). Target cells (T) were then mixed with PBMC effector cells (E) at an effector/target (E/T) ratio of 10:1 in 96-well V-bottom plates (Corning, Corning, NY, USA). Pooled mice sera (1:1000) were added to the appropriate wells. The plates were subsequently centrifuged for 1 min at 300× *g* and incubated at 37 °C, 5% CO_2_ for 5 to 6 h before being fixed in a 2% PBS-formaldehyde solution. ADCC was calculated as previously reported [54] using the formula: [(% of GFP+ cells in Targets plus Effectors) − (% of GFP+ cells in Targets plus Effectors plus sera)]/(% of GFP+ cells in Targets) × 100 by gating on infected lived target cells. All samples were acquired on an LSRII cytometer (BD Biosciences, Franklin Lakes, NJ, USA) and data analysis was performed using FlowJo (Version 10, Tree Star, Ashland, OR, USA).

### 2.5. Cell Surface Staining

For cell surface staining against cells infected with ADA-Env-based NL4.3 GFP viruses containing intact (WT) or defective *nef* and *vpu* genes (N-U-), forty-eight hours post-infection, primary CD4+ T cells were stained for 30 min at 37 °C with pooled mice sera (1:1000 dilution) in PBS. Cells were then washed once with PBS and stained with 2 µg/mL anti-mouse AlexaFluor 647 (AF-647) secondary antibodies and 1:1000 dilution of viability dye AquaVivid (Thermo Fisher Scientific, Waltham, MA, USA) for 20 min in PBS at room temperature. Cells were then washed with PBS and fixed in a 2% PBS-formaldehyde solution. All samples were acquired on an LSRII cytometer (BD Biosciences, Franklin Lakes, NJ, USA) and data analysis was performed using FlowJo (Version 10, Tree Star, Ashland, OR, USA).

### 2.6. Competition ELISA

ELISA plates for competition ELISA were prepared as above. Biotinylated A32 and N5-i5 were generated using the EZ-link Sulpho-NHS biotin kit (Thermo Fisher Scientific, Waltham, MA, USA). The half-max binding was then calculated against ID2 and a 2× concentration of half-max A32 or N5-i5 was mixed 1:1 with a 2x concentration of diluted sera. These samples were plated alongside an internal control of unbiotinylated control A32 or N5-i5 (dilution curve starting at 10 µg/mL) to determine 100% competition and naïve pre-bleed sera to determine 0% competition. Following incubation overnight, plates were washed and a 1:1000 dilution of ExtrAvidin AP (Sigma Aldrich, St. Lewis, MO, USA) was added to the plates for 1 h at room temperature. Plates were developed and read as above. GraphPad Prism was used to calculate the % competition of each sera sample by normalizing results to the 100% and 0% internal plate controls. All sera were assayed in triplicate for each individual mouse, displayed is mean with SEM for each immunization group.

### 2.7. Statistics

All statistical analysis was carried out using GraphPad Prism. Statistical significance was determined using a one-way ANOVA with Tukey’s multiple comparisons. Significance values are indicated as * *p* < 0.05, ** *p* < 0.01, *** *p* < 0.001, and **** *p* < 0.0001.

## 3. Results

### 3.1. The Overall ID2 IgG Titer in Mice Sera Increases with ID2 Immunization Dose

**Figure 1:**
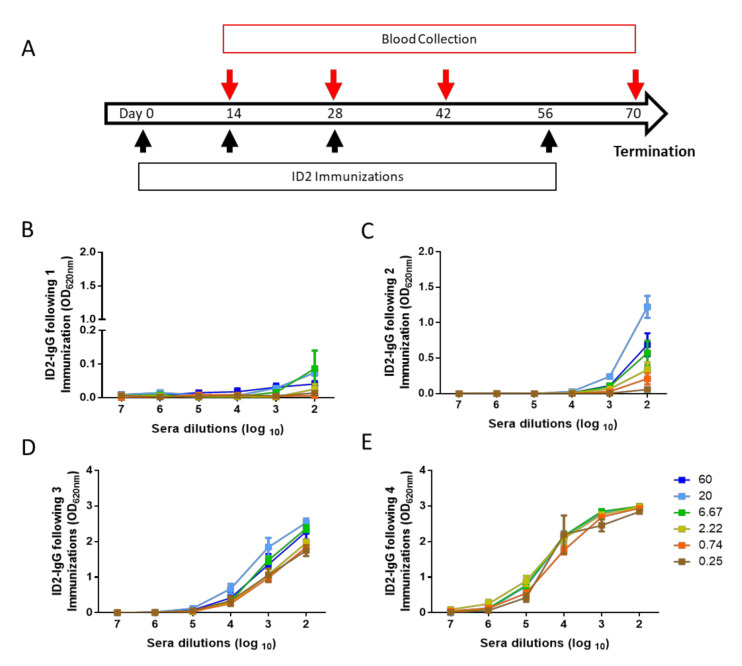
Kinetics and titer of ID2-specific IgG. Kinetics and titers of ID2-specific IgG induction over the course of ID2 + GLA-SE immunization using the vaccination scheme shown in (**A**). Throughout the immunization protocol, sera were collected two weeks post vaccination for each time point and analyzed for the induction of IgG against ID2 in an ELISA format. Sera were analyzed 2 weeks following (**B**) one immunization, (**C**) two immunizations, (**D**) three immunizations, and (**E**) the fourth and final immunization. *n* = 6 mice for each group, sera were evaluated for each mouse in triplicate and displayed as mean ± SEM.

To investigate the immunogen dose effect on the induction of ID2-specific immunoglobulin, we immunized groups of 6 BALB/c mice (three female, three male) via the intraperitoneal route with ID2 doses ranging from 0.25 to 60 µg in GLA-SE adjuvant as previously described [52] and outlined in Figure 1A. GLA-SE adjuvant (a stable oil-in water emulsion containing a TLR-4 agonist adjuvant developed by the Infectious Disease Research Institute) was used because it provided the most specific and functional response to Cluster A [52]. Sera were collected 2 weeks post immunization, throughout the course of the 4 immunizations and assayed for ID2-specific total IgG titers using a secondary antibody that detects all IgG isotypes, in addition to cross-adsorbing IgA and IgM. Sera collected two weeks following the first immunization with ID2 revealed that a small amount of specific IgG had been generated, most obviously in the groups of mice dosed with 20 and 6.67 µg of ID2 (Figure 1B). Following two immunizations, the largest titer of ID2-IgG was induced following the 20 μg dose with all other doses, except the lowest—0.25 μg—resulting in detectable ID2 IgG (Figure 1C). After three immunizations, all dosing groups had detectable specific titers, with 20 μg still inducing the highest amounts (Figure 1D). By the termination of the experiment all doses above 2.2 μg resulted in similar titers, with the two lowest doses inducing slightly lower amounts of ID2-specific antibody (Figure 1E).

### 3.2. Higher ID2 Immunization dose Fails to Induce Sera Antibodies with Potent ADCC Activity

**Figure 2:**
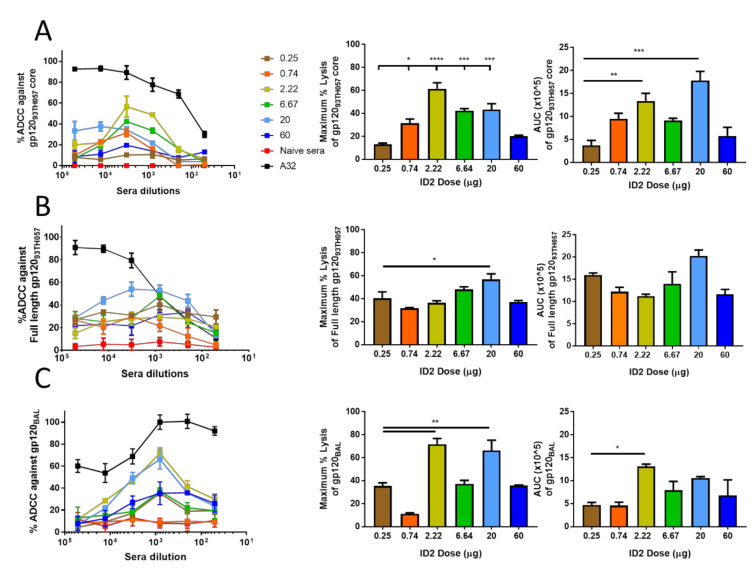
RFADCC capabilities of sera from ID2 immunized mice. The RFADCC potential of pooled sera from ID2 immunized mice was assayed against a number of targets. (**A**) RFADCC against gp120_93TH057_ core coated (**B**) full-length gp120_93TH057_ and (**C**) gp120_BaL_ coated EGFP-CEN-NKR-CCR5SNAP target cells. The left panels show the % RFADCC over a number of sera of A32 positive control antibody concentrations over a 4-fold dilution. The middle panels display the maximum % lysis against each target and the right panels display the overall area under curve (AUC) for each immunization group. Sera were pooled for each dosing group and analyzed 2 weeks following the final immunization. *n* = 6 mice for each group, pooled sera were evaluated in triplicate and displayed as mean ± SEM. Max % lysis and AUC were analyzed for statistical significance using a one-way ANOVA with Tukey’s test for multiple comparisons. Significance values are indicated as * *p* < 0.05, ** *p* < 0.01, *** *p* < 0.001, and **** *p* < 0.0001.

The ID2 immunogen was designed to exclusively induce Cluster-A-specific nnAbs capable of potent ADCC against virus-coated target cells where CD4-triggered HIV-1 envelope targets are still available [18,31,51]. To test if the induced ID2 antibody titers correlated with the ADCC activities of the sera, we analyzed terminal sera from all dosing groups for ADCC activity against gp120 targets bound to GFP-CEM-NKR-CCR5SNAP cells expressing CD4 and CCR5 in a rapid fluorometric antibody dependent cellular cytotoxicity (RFADCC) assay [56] as well as against WT and Nef-VPU- virally infected target cells [26] (Figure 2 and Figure S1A). Of note, both assays express Env in the CD4-bound conformation, which is a prerequisite for anti-cluster A antibodies to engage with its epitopes.

In the RFADCC assay, we used three different gp120 variants: gp120_93TH057_ core stabilized in the CD4-bound conformation by the same C^65^-C^115^ disulfide bond used for ID2 [51] (Figure 2A), full-length, wild-type gp120_93TH057_ (Figure 2B) and full-length, wild-type gp120_BaL._ (Figure 2C). The gp120_93TH057_ variants represent ID2 clade-matched targets (ID2 immunogens consists of clade AE_93TH057_ sequence [51]), while gp120_BaL_ is a clade mismatched variant from clade B HIV-1. Interestingly, in contrast to the trends observed of ID2 titers, where immunization doses in a range of 2.22–60 μg induced similar levels of total ID2-specific IgG at the immunization termination, the ADCC potency of mouse sera in the RFADCC assays differed significantly. As shown in Figure 2A, RFADCC against gp120_93TH057_ core coated cells showed a clear dose–response curve when maximal ADCC and AUC were plotted against the dose of ID2 with high and low immunogen doses resulting in poorer ADCC (Figure 2A). This indicates that it may be possible to improve in vivo ADCC effector function by first optimizing the dose of immunogen. Interestingly, the highest dose of immunogen did not result in the best ADCC and in fact resulted in the second lowest maximal lysis (20.15%) after the 0.25 µg dose (13%) with a similar pattern for the plots of area under the curve. The best ADCC as measured by percent (%) lysis was observed for the 2.2μg dose, but the most potent (the max lysis at lower sera dilutions and therefore the largest AUC) was observed for the 20 μg immunization of ID2 (Figure 2A). We then assayed the ADCC potential against full-length gp120 of the same clade—Clade AE (Figure 2B)—or against a different clade—BaL gp120—Clade B (Figure 2C). Similar to our positive control A32, a prototype antibody of Cluster A region, mouse sera showed disparity between the two clades of gp120 with ADCC activity being slightly more potent against clade AE than B, as demonstrated by higher AUC values for clade AE (Figure 2B,C). The dose–response curve of sera tested was not as clear as with the full-length gp120 target models but there was still a benefit from a dose lower than 60 µg with the same clade benefiting the most from a 20 µg dose of ID2 and the cross clade from a 2.2 µg ID2 dose.

Finally, we tested if Abs from ID2 immunized sera were able to bind and mediate ADCC against primary CD4^+^ T cells infected with cross-clade ADA-Env-based NL4.3 viruses containing intact or defective *nef* and *vpu* genes. As predicted, we observed low binding against targets infected with wild-type viruses (Figure S1A), in agreement with data showing that CD4i targets are not available for Ab recognition on productively infected cells due to CD4 downregulation and expressed Env predominately present in its native “closed” conformation [14,26,28,35,57,58]. Contrastingly, cells infected with the *nef* and *vpu* deficient (N-U-) viruses, which express Env targets in their “open” CD4i conformation due to imperfect CD4 downregulation, were efficiently bound by sera from ID2 immunized animals with the highest level seen in mice immunized with 2.2 µg ID2. When ADCC at a sera dilution of 1:1000 were investigated, the higher doses of ID2 (60 and 20 µg) were found to mediate little or no killing of N-U- ADA infected cells, while those of a dose of 6.67 µg or less induced consistent specific killing of N-U- ADA infected cells (Figure S1B).

### 3.3. Higher ID2 Immunization dose Failed to Induce Sera Antibody Responses with Increased C1/C2 Epitope Specificity

**Figure 3:**
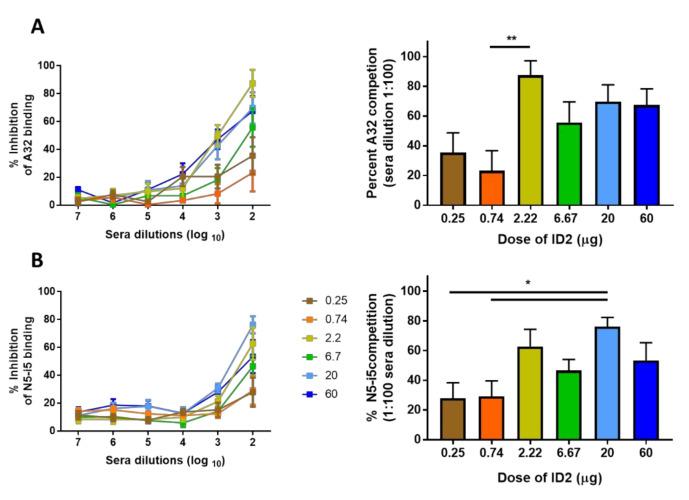
Ability of ID-2 immunized sera to compete with A32 and N5-i5 for ID2 binding. Competition ELISA to determine the level of competition of immunized sera with (**A**) A32 and (**B**) N5-i5. Sera were mixed 1:1 with a biotinylated antibody at a concentration equivalent to the half-max binding against ID2 and analyzed for % competition. Sera from each mouse were analyzed in triplicate over a 10-fold dilution beginning at 1:100 (Left panels) with % competition at 1:100 dilution displayed in right panels. *n* = 6 mice for each group, sera were evaluated for each mouse in triplicate and displayed as mean ± SEM. Statistical significance was determined using a one-way ANOVA with Tukey’s test for multiple comparisons. Significance values are indicated as * *p* < 0.05, ** *p* < 0.01.

Our analysis of functional responses in mice immunized with various doses of ID2 immunogen revealed differences in the effectiveness of Fc-effector functions of antibodies induced by different ID2 doses with the sera of mice immunized with the lower and highest ID2 dose showing the poorest ADCC in RFADCC assays. ID2 was designed to optimally present the Cluster A epitope targets; we therefore tested if antibodies with the desired epitope specificity were induced in mice immunized with different ID2 doses. We assayed the levels of sera antibodies capable of direct competition to plate bound ID2 with A32 and N5-i5, two well-studied Cluster A antibodies [59]. As shown in Figure 3, we observed the highest competition (87.4%) with A32 from sera of mice immunized with 2.22μg of ID2 and good competition (69.7 and 67.4%) for the 20 and 60 μg doses. The highest competition for N5-i5 binding reached 62.8% and 76.2% in sera of mice immunized with 2.22 and 20 μg, respectively. All together, these data indicate that the very low doses of ID2 (0.25 and 0.75 μg) are not optimal for effective induction of a Cluster-A-specific response. This points toward the possibility that the observed decreased Fc-effector function activity of the sera of mice immunized with very low doses, at least in part, is due to the poor induction of antibodies with desired Cluster A specificity. This observation could be explained by the inability to engage sufficient B-cell receptors to drive the expansion and differentiation of appropriate B cells, as described recently by [60]. Interestingly, we observed no increased potency in responses to higher ID2 immunogen, indicating that once an optimal level of B cell receptor saturation has occurred, no added benefit to antibody affinity will occur. This is in agreement with previously published data studying the effect of antigen amount which show that a higher antigen load leads to increased plasma cell differentiation but lower antibody affinity, while a lower dose of antigen leads to an increased B cell memory pool as well as antibodies of higher affinity [61].

### 3.4. Higher ID2 Immunization dose Induces IgG Isotypes with Lower Fc-Functionality

**Figure 4:**
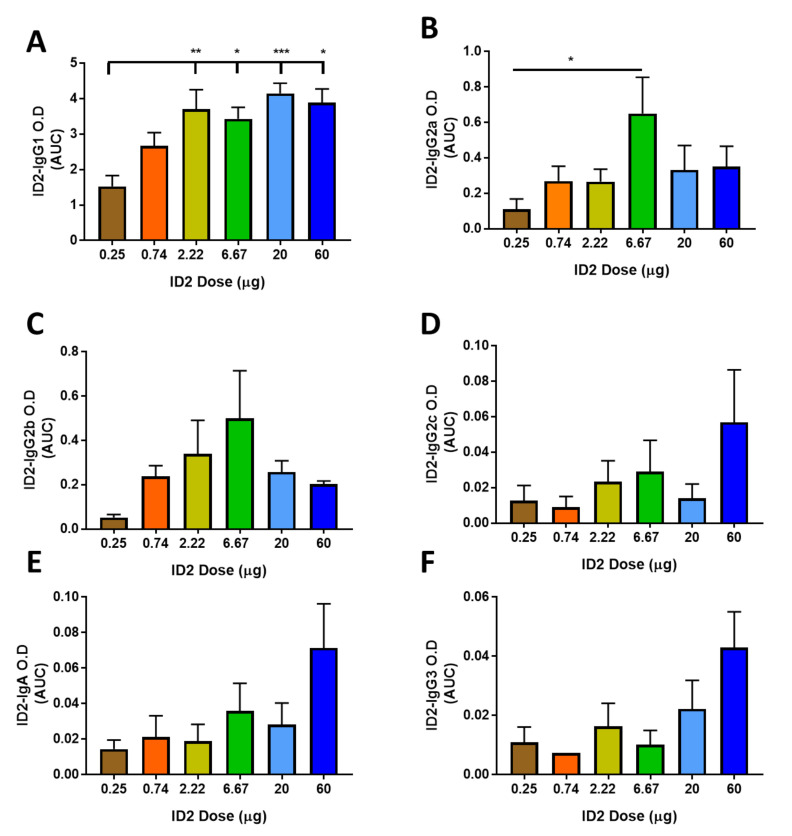
Titer of ID2-specific isotypes induced by varying doses of ID2. Titers of ID2-specific isotypes at the termination of ID2 + GLA-SE immunization. Terminal sera were collected two weeks post final vaccination and analyzed for (**A**) IgG1, (**B**) IgG2a, (**C**) IgG2b, (**D**) IgG2c, (**E**) IgA and (**F**) IgG3. *n* = 6 mice for each group, sera were evaluated for each mouse in triplicate and displayed as mean ± SEM for area under the curve (AUC) over the dilution range displayed in Figure 1. Statistical significance was determined using a one-way ANOVA with Tukey’s test for multiple comparisons. Significance values are indicated as * *p* < 0.05, ** *p* < 0.01, *** *p* < 0.001.

In Fc-effector mechanisms, an antibody plays the role of the bridge between the target and the effector cell that mediates inactivation/killing, and it is well known that the efficiency of Fc-effector activities is largely Ig isotype (class)-dependent. To analyze if immunogen dose could lead to the induction of different isotypes that differentially bind the Fcγ receptors on monocytes and result in altered RFADCC activity, we analyzed the sera at the time point of ADCC testing (at the termination) for the presence of anti-ID2-specific IgG1, IgG2a, b and c, IgG3, IgA, and IgM (Figure 4 and Figure S2). Sera were diluted over a range from 1:100–1:10^7^ and plated for ELISA analysis. The assay was done and the area under curve (AUC) calculated (Figure 4). We generated a heat map with red representing the highest amount of each isotype between dosing groups and green the lowest, numerical values representing the AUC of ID2-specific Abs over the full dilution range are also shown (Figure S2). Interestingly, while no significant differences were observed between the total ID2-specific Ig levels, the 60 µg dose resulted in an AUC of 7.33 as compared to 6.49 for the lowest dose of 0.25 µg (Figure 1 and Figure S2), we detected significant differences in the specific isotypes induced by different doses. We observed differences in the levels of ID2-specific IgG1, a murine isotype not involved in ADCC, which increased significantly with doses of ID2 over 2.2 µg and reached a maximum AUC of 4.15 following a dose of 20 μg (Figure 4A). In contrast, neither IgG2a nor IgG2b levels followed the pattern of increased specific isotype amount with increased immunogen dose. Instead, both isotypes were optimally induced at a dose of 6.67 µg, declining with lower or larger doses of immunogen. Interestingly, the levels of IgG2b, known to be involved in Fc-effector mechanisms in mice, were the highest in the sera of mice immunized with the ID2 doses in a range of 2.2–20 μg, for which we also observed the most efficient RFADCC (Figure 2). In contrast, IgG2c, IgG3 and IgA were induced at the highest levels in mice immunized with the highest 60 μg dose that performed poorly in our Fc-effector function assays. IgG3 specific for ID2 was induced in all immunization groups; however, this was at very low levels close to the level of detection for the ELISA performed. Interestingly, human IgG3 and IgA correlated with effective and poor vaccine protection, respectively, due to Fc-effector functions in RV144 [62]. Although there is not much known about Fc-dependent functionality of the mouse IgG2c, IgG3 and IgA isotype, especially in Fc-effector functions involving human PBMCs, the elevated levels of these subclasses in the sera of mice immunized with the highest ID2 that shows suboptimal Fc-effector activity points towards the negative rather than positive effect for these subclasses in the RFADCC activity of sera antibodies.

### 3.5. Isotype Specificity and Antibody Affinity in the 2.2 μg dosing Group Affects ADCC Potency

**Figure 5:**
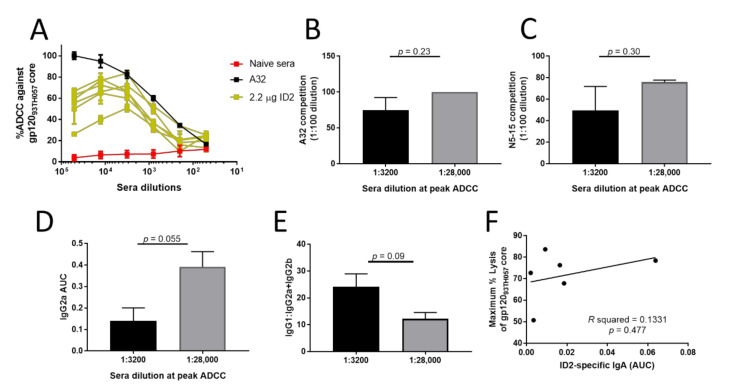
RFADCC capabilities of individual sera from 2.2 µg ID2 immunized mice. The RFADCC potential of individual sera from ID2 immunized mice was assayed against gp120_93TH057_ core coated EGFP-CEN-NKR-CCR5SNAP target cells. (**A**) RFADCC of 2.2 µg ID2 immunized mice over a broad concentration range of sera dilutions for each individual mouse. (**B**) A32 and (**C**) N5-i5 competition displayed segregated by potency of RFADCC response. (**D**) IgG2a AUC value and (**E**) ratio of IgG1:IgG2a + IgG2b as segregated by potency of RFADCC response. (**F**) Correlation of IgA to RFADCC showing R squared and *p* calculated using Pearson’s two-tailed correlation. *n* = 6 mice, individual sera were evaluated in triplicate and displayed as mean ± SEM. Statistical significance was analyzed using an unpaired *T*-test.

In order to further investigate the relationship between the epitope specificity of the induced response, i.e., Ig isotype and ADCC efficiency, we analyzed individual sera from the 2.2 µg dose group, which showed the most effective RFADCC against cells coated with stabilized gp120_93TH057_core_e_ and full-length gp120_BaL_ (Figure 2). Interestingly, when sera were analyzed individually for each mouse, the difference in potency became apparent with half of the sera samples peaking at 1:3200 and the other half at 1:28,000 (Figure 5A), showing differences in ADCC potency in response to the same immunogen dose. When the specificity of the induced antibody response was evaluated (Figure 5B,C), sera with decreased ADCC potency had more variable and an overall lower level of competition to both A32 and N5-i5 than sera with higher ADCC potency. This points toward a key role of precise epitope specificity of the induced response in Fc-effector function. In addition, the level of ID2-specific IgG2a, the murine isotype most associated with ADCC, varied between the two groups with higher IgG2a associated with increased ADCC potency (Figure 5D). This increased IGg2a also translated to a much smaller ratio of IgG1 to IgG2a + IgG2b (Figure 5E), indicating that more potent ADCC is associated with increased antibody specificity and a shift toward the induction of antigen-specific ADCC relevant isotypes in mice. Finally, in RV144, one important correlation was an inverse relationship between anti-Env IgA and protection [62]. IgA induced to the same epitope targets competed with ADCC functional IgGs of the same specificity [63]. To analyze if ID2-specific IgA could interfere with IgGs and decrease the overall ADCC activities of mouse sera immunized with 2.2 μg ID2, we performed a correlation analysis of the IgA response (AUC) with ADCC against gp120 core_93TH057_ (Figure 5F) and saw no correlation between the level of induced ID2 IgA and RFADCC.

## 4. Discussion

Existing evidence shows an important role of Cluster A epitopes in ADCC to natural infection [64,65,66,67] as well as in protective effect of the RV144 vaccine [42,43]; however, it is unknown if a non-neutralizing ADCC response to this region alone can confer protection. Passive transfer experiments in NHP models with nAbs of variable specificities are inconclusive. The three NHP studies using nnAbs, including the C1/C2 specific mAb A32, failed to provide protection, although evidence of the impact of the antibody on the transmitted virus was clear in all cases [68,69,70]. A passive transfer study with rigorously tested nnAb doses or protection studies with vaccine-induced nnAbs is required to definitively address a role of Fc-mediated effector responses of nnAbs in HIV-1 protection.

We previously developed ID2, an immunogen that stably incorporates the C1/C2 epitopes within a 25 kDa unit of HIV-1 gp120 stabilized in CD4-bound conformation [51,52]. ID2 has the potential to be developed into a vaccine candidate that could be used to test if a vaccine-induced non-neutralizing polyclonal response to C1/C2 epitopes protects against HIV infection. If done, such studies will provide a definitive answer about the role of C1/C2 targets in HIV-1 protection. To continue with ID2 optimization, we performed an immunogen dose study in BALB/c mice to test if the quantity and quality (as measured by functional activity) of the induced immune response can be modulated by dose delivered by intraperitoneal (IP) in four immunizations. To factor in the potentially vital influence of antibody amount and isotype switching, we designed an immunization protocol over a wide dose range of immunogen (0.25–60 μg) to provide a polyclonal, species-matched investigation of antibody induction and ADCC effectiveness.

Our studies clearly indicate that the functional response to ID2 is ‘fine-tuned’ by immunogen dose. While ADCC potency of sera did not directly correlated with the level of induced ID2-specific antibodies, there was clear evidence for a correlation between ADCC and the ID2-specfic isotype pattern. The specific interaction of antibodies with the target is mediated through the Fab of the antibody and the interaction with the effector cell through the Fc part of the antibody that binds the Fc receptor expressed at the effector cell surface [53]. Fc-effector mechanisms therefore heavily depend on engagement of antibody Fc regions into effective complexes with Fcγ-receptors at the effector cell surface. There is growing evidence describing how Ig isotypes interact with Fcγ receptors expressed at the surface of human effector cells and how the Ig isotype is linked to the effectiveness of particular Fc-effector function (e.g., ADCC, ADCP etc.) [71,72,73]. By contrast, there is very little information about murine Ig isotype involvement in Fc-effector processes. For murine antibodies, IgG2a (equivalent to human IgG3), IgG2b (equivalent to human IgG1) and IgG3 are known to be involved in Fc-effector mechanisms [74]. In addition, both murine IgG2a and IgG3 have been shown to induce ADCC and generate IFNγ in the presence of human monocytic cells, indicating the Fc region of both isotypes are able to bind and activate human Fcγ receptors on human monocytes [75], IgG2a is thought to play the role of human IgG3 as it displays the highest affinities to FcγRIV, the primary receptor involved in ADCC in mice [76]. This is of particular importance for our studies since all ADCC measurements were carried out using human PBMCs [56] in an RFADCC assay know to heavily rely on monocytes [77].

We determined that optimal dosing conditions were required to induce an isotype repertoire able to mediate potent ADCC in the absence of isotypes known to negatively affect ADCC. This indicates that, in contrast to vaccination strategies designed for the induction of a neutralizing antibody response, a vaccine based solely on, or including components of, a Fc-effector antibody response will require careful investigation of the immunization protocol to include a dose that allows for the required class switching and fine-tuning of the B cell response. This will also require careful investigation of new vaccine candidates in each animal model before the move to humans, as there is growing evidence of significant interspecies differences (human versus mice, human versus non-human primates, etc.) in the individual components of the immune system involved in Fc-effector mechanisms. These include differences in the isotype/subclass/allele repertoire of immunoglobulins and FcRs that can confound outcomes of such studies [78,79].

## Supplementary Materials

The following are available online at https://www.mdpi.com/2076-2607/8/10/1490/s1, Figure S1: Binding and ADCC capabilities of sera from ID2 immunized mice against WT and N-U-ADA infected target cells. Figure S2: Mean Area under curve values for all tested ID2-specific Isotypes.
