# Supplementary material for: Effects of gp120 Inner Domain (ID2) Immunogen Doses on Elicitation of Anti-HIV-1 Functional Fc-Effector Response to C1/C2 (Cluster A) Epitopes in Mice
Source: Microorganisms. 2020 Sep 28;8(10):1490. doi: 10.3390/microorganisms8101490 (PMC7650682; doi:10.3390/microorganisms8101490)
Supplement: Supplementary file 1 [file microorganisms-08-01490-s001.zip › microorganisms-920620 R1 supplementary reformed/Sup legends.docx]

**Figure S1. Binding and ADCC capabilities of sera from ID2 immunized mice against WT and N-U- ADA infected target cells. (A)** The binding of pooled sera from ID2 immunized mice was assayed against WT of nef-vpu- (N-U-) ADA infected target cells. **(B)** ADCC of pooled sera from ID2 immunized mice was assayed against WT of nef-vpu- (N-U-) ADA infected target cells. Data is displayed as N-U- minus WT background ADCC. *n* = 6 mice for each group, pooled sera was evaluated in triplicate and displayed as mean ± SEM.

**Figure S2. Mean Area under curve values for all tested ID2-specific Isotypes.** Heat map displaying the mean AUC values for all ID2-specific isotypes tested. Red defines the highest value within the isotype group and green the lowest. *n* = 6 mice for each group, pooled sera was evaluated in triplicate and displayed as mean.
