# Supplementary figures and images for: Effects of gp120 Inner Domain (ID2) Immunogen Doses on Elicitation of Anti-HIV-1 Functional Fc-Effector Response to C1/C2 (Cluster A) Epitopes in Mice
Source: Microorganisms. 2020 Sep 28;8(10):1490. doi: 10.3390/microorganisms8101490 (PMC7650682; doi:10.3390/microorganisms8101490)

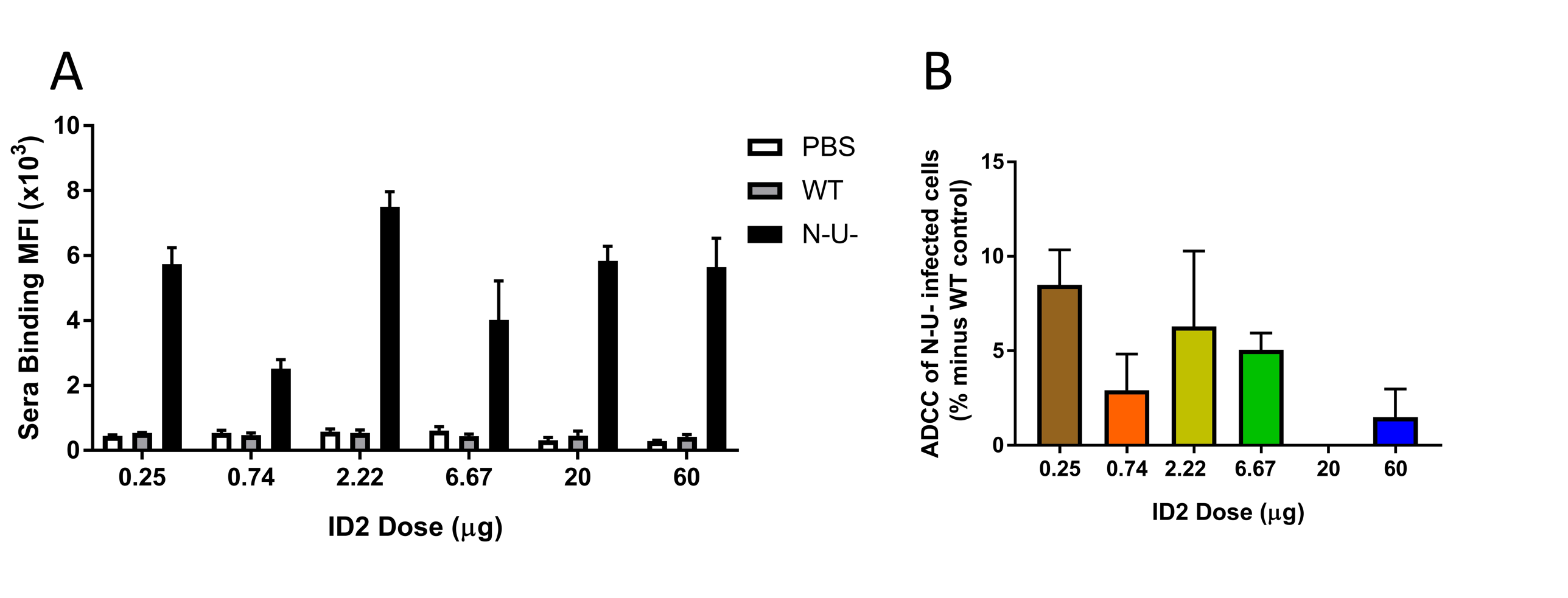

Supplement: Supplementary file 1 [file microorganisms-08-01490-s001.zip › microorganisms-920620 R1 supplementary reformed/Figure S1.jpg]

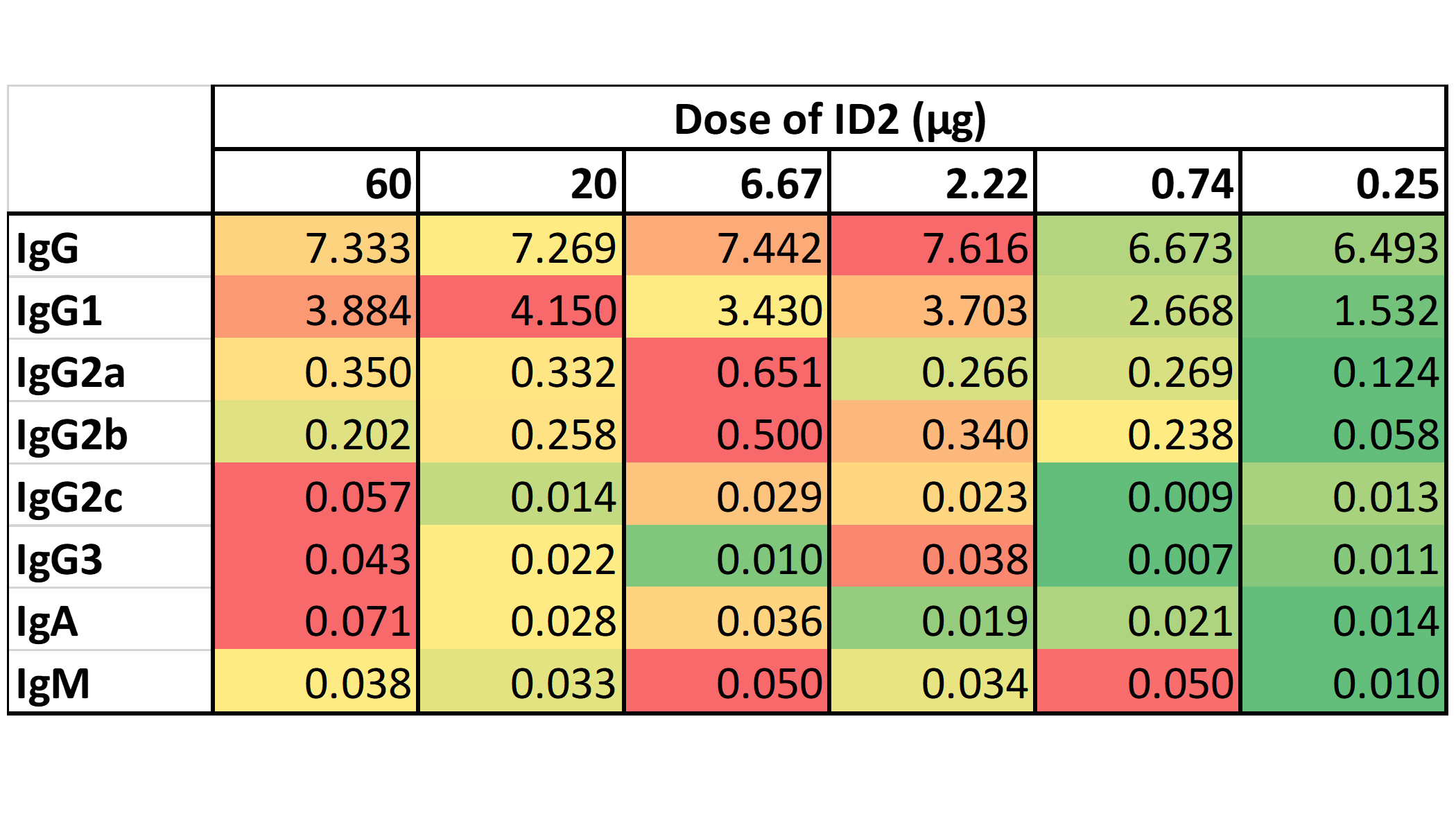

Supplement: Supplementary file 1 [file microorganisms-08-01490-s001.zip › microorganisms-920620 R1 supplementary reformed/Figure S2.tif]
